# Repeated brief isoflurane anesthesia during early postnatal development produces negligible changes on adult behavior in male mice

**DOI:** 10.1371/journal.pone.0175258

**Published:** 2017-04-05

**Authors:** Marko Rosenholm, Emmi Paro, Hanna Antila, Vootele Võikar, Tomi Rantamäki

**Affiliations:** 1 Laboratory of Neurotherapeutics, Division of Physiology and Neuroscience, Faculty of Biological and Environmental Sciences, Department of Biosciences, University of Helsinki, Finland; 2 Neuroscience Center, Helsinki Institute of Life Science, University of Helsinki, Helsinki, Finland; Massachusetts General Hospital, UNITED STATES

## Abstract

Brain development is a complex process regulated by genetic programs and activity-dependent neuronal connectivity. Anesthetics profoundly alter neuronal excitability, and anesthesia during early brain development has been consistently associated with neuroapoptosis, altered synaptogenesis, and persistent behavioral abnormalities in experimental animals. However, the depth, and even more the duration and developmental time point(s) of exposure to anesthesia determine the neuropathological and long-term behavioral consequences of anesthetics. Here, we have investigated adulthood phenotypic changes induced by repeated but brief (30 min) isoflurane anesthesia delivered during two distinct developmental periods in male mice. A set of animals were subjected to anesthesia treatments at postnatal days 7, 8 and 9 (P7-9) when the animals are susceptible to anesthesia-induced neuroapoptosis and reduced synaptogenesis. To control the potential influence of (handling) stress, a separate group of animals underwent repeated maternal separations of similar durations. Another set of animals were exposed to the same treatments at postnatal days 15, 16 and 17 (P15-17), a developmental time period when anesthetics have been shown to increase synaptogenesis. Starting from postnatal week 9 the mouse phenotype was evaluated using a battery of behavioral tests that assess general locomotor activity (home cage activity, open field), learning and memory (water maze) and depression- (saccharin preference, forced swim test), anxiety- (light-dark box, stress-induced hyperthermia) and schizophrenia- (nesting, prepulse inhibition) related endophenotypes. Apart from mild impairment in spatial navigation memory, exposure to anesthesia treatments during P7-9 did not bring obvious behavioral alterations in adult animals. Importantly, maternal separation during the same developmental period produced a very similar phenotype during the water maze. Mice exposed to anesthesia during P15-17 showed mild hyperactivity and risk-taking behavior in adulthood, but were otherwise normal. We conclude that significantly longer administration periods are needed in order for early-life repeated exposures to anesthetics to produce behavioral alterations in adult mice.

## Introduction

Anesthetics produce concentration-dependent general anesthesia (unconsciousness, insensateness, analgesia and amnesia). General anesthetics are widely used in various medical practices requiring (surgical) anesthesia. Along with the continuous advances in modern preclinical invasive techniques (e.g. *in vivo* imaging), the use of anesthetics is steadily growing in biomedical research involving immature animals. Most general anesthetics, such as volatile halogenated hydrocarbons (e.g. isoflurane, sevoflurane), have relatively short half-lives which allows for rapid recovery following drug discontinuation. Pharmacologically anesthetics primarily act through facilitating GABA_A_ receptor function and/or dampening the glutamatergic NMDA (*N*-methyl-*D*-aspartate) receptor activity [1]. Glutamate is the principle excitatory neurotransmitter in the central nervous system. GABA_A_ receptors are chloride channels activated by GABA (gamma-aminobutyric acid), and are responsible for inhibitory synaptic transmission in the adult brain.

Brain development is a highly complex and dynamic process that is guided by genetic and epigenetic mechanisms, as well as intrinsic and extrinsic neuronal activity [2–4]. Neuronal connections are wired and fine-tuned through optimally balanced activity-dependent mechanisms, and this process is very vulnerable to disturbances. Exposure to anesthetics—particularly during the first postnatal weeks–have been shown to produce neuropathological changes, including neuroapoptosis and synapse loss and persistent behavioral abnormalities in rodents [5]. Interestingly, exposure to anesthetics during postnatal days 15–25 is shown to facilitate synaptogenesis in rodents [6–8], but the underlying neurobiological basis and ultimate long-term functional and behavioral consequences (and translational meaning) of this remain obscure. Altogether these emerging preclinical observations have generated awareness and concern regarding the clinical safety of anesthetics in pediatric patients and pregnant women [9]. Indeed, some studies indicate that young children are more susceptible to anesthesia-induced behavioral impairments such as learning disabilities and cognitive problems [10]. However, it is difficult to differentiate whether the observed changes stem from the treated condition itself, or from the actual anesthetic [10,11].

The clinical relevance of animal data is further complicated by marked species-specific differences in developmental processes, particularly the length of developmental time windows [12]. Indeed, the majority of animal studies have investigated the effects of proportionally long anesthesia treatments, however the depth and duration of anesthesia do significantly correlate with neuropathological and behavioral impairments [12–17]. Most animal studies have utilized a 2–6 hour anesthesia exposure time frame, which likely equates to several days of anesthesia in humans. Although similar anesthesia exposure brings acute neuropathological alterations even in non-human primates [18]; it remains unknown whether this leads to persistent functional or behavioural impairments. Importantly, a recent animal study investigated the effects of a single 30-minute anesthesia, delivered to rats at P7, on neuroapoptosis and synaptogenesis during development and behavior in adulthood [8]. This brief anesthesia increased acute neuropathological alterations, but these changes were not associated with prominent behavioral abnormalities in adult animals. Notably, some animal studies indicate that repeated—even relatively short—exposure to deep anesthesia during different stages of postnatal development lead to more prominent neuropathological and behavioral alterations when compared to a single treatment [19,20]. Such observations are translated in clinical studies that show repeated, but not single, anesthesia in children younger than 4 years old to develop learning disabilities later in life [10,21]. The purpose of this study was to evaluate the long-term behavioral consequences of repeated brief isoflurane anesthesia–i.e. consecutive but transient disruptions of excitation-inhibition balance–in mice when administered at two distinct developmental periods: postnatal days 7, 8 and 9 (P7-9) or postnatal days 15, 16 and 17 (P15-17).

## Materials and methods

### Animals and treatments

**Fig 1:**
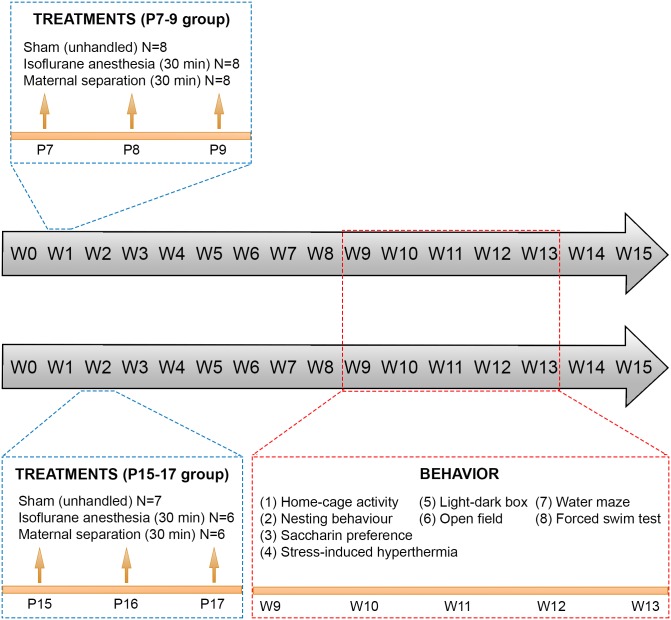
Experimental time-line. At postnatal days 7, 8 and 9 (P7-9) or 15, 16 and 17 (P15-17) male mice were subjected to 30 min maternal separation or 30 min isoflurane anesthesia, or were left unhandled (sham). Animals were weaned at P21 (week 3; W3). Beginning from age of 9-weeks animals were subjected to a behavioral phenotyping battery consisting of circadian activity, nest construction and saccharin preference (InfraMot monitoring system), light-dark box, open field, prepulse inhibition (PPI), water maze, stress-induced hyperthermia and forced swim test. N = 6-8/group.

Adult female C57BL/6JRccHsd mice were bred with C57BL/6JRccHsd male mice in two cohorts to obtain 24 and 19 male pups for the early-life manipulations performed at postnatal days (P) 7–9 and P15-17, respectively. Specifically, the mouse pups were subjected to 30-minute isoflurane (Vetflurane^®^, Virbac) anesthesia treatments or mere 30-minute maternal separations (P7-9: N = 8/group; P15-17: N = 6/group) (Fig 1). Animals subjected to maternal separation were placed in a separate mouse cage covered with bedding material and constantly kept warm with a heat pad. Deep isoflurane anesthesia was delivered as previously described for adult mice [22]: induction with 4% isoflurane for 2 minutes, followed by maintenance with 2% isoflurane for 30 minutes (oxygen flow: ~400 ml/min). Body temperature was maintained with a heat pad during the treatments. After a short recovery period, the mice were placed back into their home cage. Sham male mice (P7-9: N = 8, P15-17: N = 7) (and female littermates) were kept unhandled in their home cage. General well-being and weight gain of the animals were monitored regularly. At P21 the male mice were weaned from their dams and group housed until behavioral testing. Female mice were not part of the experimental design and they were euthanized at this point. Only male mice were used because of their reportedly higher susceptibility to the deleterious effects of early-life anesthesia in comparison to females [23,24]. During the treatments animals were identified by tail marks. For longitudinal identification of the animals, earmarks were made after the treatments.

The animal experiments were carried out according to the guidelines of the Society for Neuroscience and were approved by the County Administrative Board of Southern Finland (License: ESAVI/10527/04.10.07/2014). Animals were maintained in the animal facility at the University of Helsinki (F-building, Viikki), Finland, under standard laboratory conditions (21°C, 12-hour light-dark cycle, lights on at 6 AM) with free access to food and water. The mice were housed in individually ventilated plastic cages (groups of 2–6 mice/cage; Mouse IVC Green Line–overall dimensions 391 x 199 x 160 mm, floor area 501 cm2; Tecniplast, Italy) with half of the cage covered by wire bar food hopper. Air inlet and outlet valves were located in the cage lid, on top of the cage.

### Behavioral methods

Behavioral phenotyping was started at the age of 9 weeks in the order presented below (Fig 1). The behavioral battery was designed in a way resembling our previously described behavioral phenotyping battery validated for mutant mice [25]. The experimenters were blinded to the previous treatments of the mice throughout the behavioral testing.

#### Circadian activity

The InfraMot system (TSE, Germany) was used for registering total activity of a single-housed animal under any lighting condition as measured by the displacement of body heat over time. The mice were housed in Type II cages (267 mm x 207 mm x 140 mm) with bedding material (aspen chips, Tapvei), with the sensor assembly mounted on the cage cover. The recording continued for 7 days.

#### Nest construction

Following one-day assimilation, nest construction was assessed in single cages containing the InfraMot system assembly. One hour before the dark phase a nestlet comprised of 2.5 g compressed cotton (Ancare, Bellmore, NY) was added to the cage. After 12 h the nests were assessed on a rating scale of 1–5 [26]: 1 = Nestlet >90% intact, 2 = Nestlet 50–90% intact, 3 = Nestlet mostly shredded but no identifiable nest site, 4 = identifiable but flat nest, 5 = crater-shaped nest.

#### Saccharin preference

Mice were trained to drink tap water from two 15 ml Falcon tubes (with cut tip; placed side-by-side) for 3 days; after which water from one tube was replaced with 0.1% saccharin. Preference for sweet solution was recorded during two 24-hour sessions (the positions of the tubes changed between the sessions). The test was performed at the InfraMot system.

#### Light-dark box

The test was carried out in an open field arena (30 cm x 30 cm, Med Associates, St. Albans, VT) equipped with infrared light sensors detecting horizontal and vertical activity. The dark insert (non-transparent for visible light) was used to divide the arena into two halves, an opening (a door with a width of 5.5 cm and height of 7 cm) in the wall of the insert allowed animal’s free movement from one compartment to another. Illumination in the center of the light compartment was ~550 lx. Animals were placed in the dark compartment and allowed to explore the arena for 10 minutes. Distance traveled, number of rearings, and time spent in different compartments were recorded.

#### Open field

The mice were released in the corner of a novel open field arena (30 cm x 30 cm, white floor, Med Associates). Horizontal and vertical activity was recorded during a 30-minute trial (light intensity ~150 lx). Peripheral zone was defined as a 6 cm wide corridor along the wall to define the time spent in the central area of the arena.

#### Prepulse inhibition

Mice were enclosed in a transparent plastic tube (Ø 4.5 cm, length 8 cm) that was placed in the startle chamber (Med Associates) with a background white noise of 65 dB and left undisturbed for 5 minutes. Testing was performed in 12 blocks of 5 trials with five trial types being applied. One trial type consisted of a 40-ms, 120-dB white noise acoustic startle stimulus (SS) presented alone. In the remaining four trial types the startle stimulus was preceded by an acoustic pre-pulse stimulus (PPS). The 20-ms PPS contained white noise bursts of 68, 72, 76 and 80 dB. The delay between onset of PPS and SS was 100 ms. The 1^st^ and 12^th^ block consisted of SS-alone trials. In remaining blocks the SS and PPS+SS trials were presented in a pseudo-randomized order such that each trial type was presented once within a block of 5 trials. The inter-trial interval ranged between 10 and 20 seconds. The startle response was recorded for 65 ms starting with the onset of the startle stimulus. The maximum startle amplitude recorded during the 65-ms sampling window was used as the dependent variable. The startle response was averaged over 10 trials from blocks 2–11 for each trial type. The pre-pulse inhibition for each PPS was calculated by using the following formula: 100-[(startle response on PPS+SS trials / startle response on SS trials) x 100].

#### Water maze

The system consisted of a white circular swimming pool (Ø 120 cm) and an escape platform (Ø 10 cm) submerged 5 mm under the water surface located in the centre of one of four quadrants. The animals were placed into the water maze in random positions facing the wall. The time to reach the escape platform (maximum time 60 s) and the total distance swam were measured in each trial. In addition, thigmotaxis, the time spent swimming within the outermost ring of the pool (10 cm from the wall), was measured. Two training blocks consisting of three trials each were conducted daily. The interval between trials was 4–5 minutes, and between training blocks about 5 hours. The hidden platform remained in a constant location for 3 days (6 initial training sessions) and was then moved to the opposite quadrant for 2 days (4 reverse training sessions). The probe trials were conducted approximately 18 h after the last initial and reverse training sessions. The mice were allowed to swim in the maze for 60 seconds without the platform available. Spatial memory in the probe trials was estimated by preference of swimming in the trained region (imaginary circular area of Ø 30 cm, around the previous platform location) over swimming in corresponding regions within the three other quadrants. The mice were video-tracked using the Noldus EthoVision XT 10 system (Noldus Information Technology, Wageningen, The Netherlands).

#### Forced swim test

The mouse was placed for 6 minutes in a glass cylinder (Ø 18 cm, height 25 cm) filled with water at 23 ± 1°C to the height of 15 cm. The time of immobility (passive floating, when the animal was motionless or doing only slight movements with tail or one hind limb, whereas the animal was judged to be active when struggling, climbing or swimming using all four paws) was measured in 2-minute intervals. The animals were tracked using the Noldus EthoVision XT 10 system (Noldus Information Technology, Wageningen, The Netherlands) during the forced swim test and immobility was automatically detected by the software.

### Statistical analyses

Unless otherwise stated, the results are represented as mean ± SEM (standard error of mean). For statistical analysis, two-way analysis of variance (ANOVA) or two-way ANOVA on ranks (non-normally distributed data: nesting behavior) were used. *Post hoc* analysis was conducted with Newman-Keuls test. Statistically significant p value was set to ≤ 0.05.

## Results

Male mouse pups were subjected to 30-minute isoflurane anesthesia or maternal separation on three consecutive days at either P7-9 or P15-17, or were left unhandled (sham groups) (Fig 1). The pups recovered rapidly from anesthesia and were placed immediately back to their home cage. Weight gain was routinely monitored and found not to be influenced by any of the treatments (data not shown). At P21 the animals were weaned and group housed. At the age of 9 weeks we performed behavioral testing battery to assess general locomotor activity, anxiety-, depression- and schizophrenia related phenotypes, and spatial and recognition memory (Fig 1). The behavioral battery used for these experiments was previously validated in our laboratory for mutant mice [25]. Notably, maternal separation in mice has not been shown to produce consistent or convincing alterations in schizophrenia-, anxiety- and depression-related or cognitive endophenotypes in adulthood [27–29].

**Fig 2:**
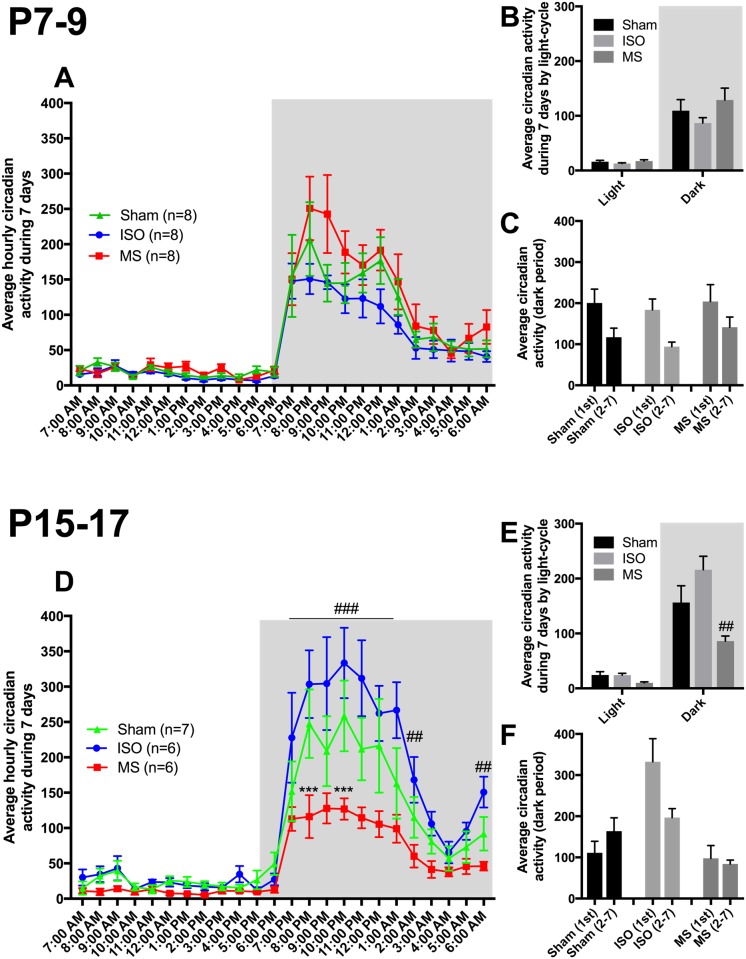
Animals exposed to repeated brief isoflurane anesthesia or maternal separation at postnatal days 15–17 show varying circadian activity at adult age. Hourly average circadian activity during 7-day monitoring (**A, D**), average circadian activity during different light-cycles (**B, E**) and average circadian activity during the first dark period in comparison to remaining dark periods (**C, F**). Lights off (active period; grey) during 6:00 PM– 6:00 AM. Abbreviations: ISO, isoflurane; MS, maternal separation. ***<0.001 two-way ANOVA followed by Newman-Keuls *post hoc* test MS vs. Sham. ###<0.001, ##<0.01 two-way ANOVA followed by Newman-Keuls *post hoc* test ISO vs. MS. N = 6-8/group.

Average hourly circadian activity during the 7-day recording period is shown in Fig 2. No significant differences in circadian activity were observed between the groups that were exposed to the treatments at P7-9 (Fig 2A and 2B). However, maternal separation and isoflurane treatments during P15-17 produced contrasting alterations on the animals’ activity at the adult age: while isoflurane treated animals showed an overall increase in activity during the dark phase, animals subjected to maternal separation displayed a contrasting phenotype (Fig 2D and 2E). The activity of the maternal separation group was strongly reduced, particularly during the early phases of dark period when compared to the sham group (Fig 2D and 2E). No significant differences were observed during the light (“inactive period” in diurnal animals) phase, although animals exposed to maternal separation tended to be more immobile during this period (Fig 2D and 2E). To evaluate whether animals showed increased locomotor activity on the first day after transfer to single-housing, we compared the activity of the animals during the first night (as measured by the InfraMot system) to the overall activity of the remaining six nights (Fig 2C and 2F). All the mice from P7-9 cohort showed more locomotor activity during the first active period compared to the remaining 6 days. Unexpectedly, the P15-17 sham and maternal separation groups did not show more locomotor activity during the first active period. However, the “hyperactivity” phenotype of animals exposed to isoflurane at P15-17 was already seen as pronounced locomotor activity during the first active period.

**Fig 3:**
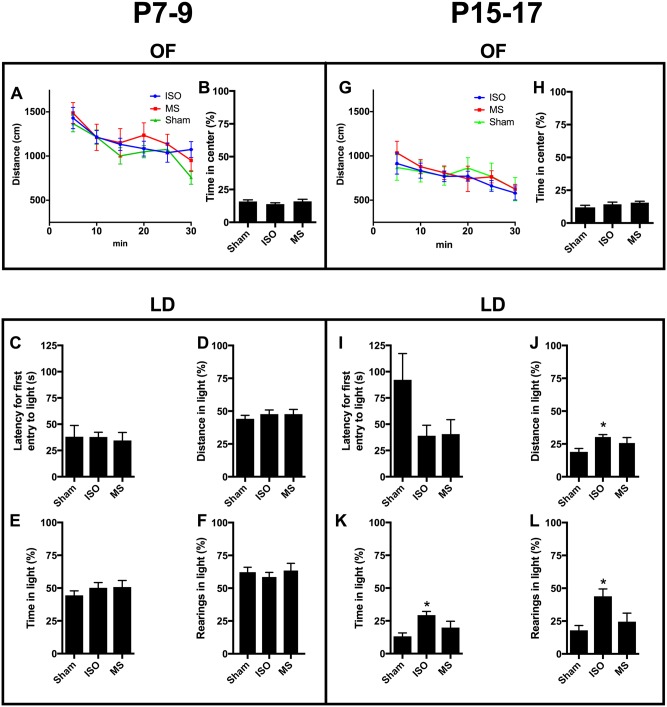
Early-life exposures to three consecutive and brief isoflurane anesthesias at postnatal days 15–17 produce mild long-lasting decrease in anxiety-related behavior. Distance travelled (and habituation) (**A, G**) and time in center (**B, H**) in a novel open environment (open field test). Latency to light (**C, I**), relative distance travelled (**D, J**), distance in light (**E, K**) and rearings in light (**F, L**) in the light-dark box test. Abbreviations: ISO, isoflurane; MS, maternal separation; OF, open field test; LD, light-dark box test. *<0.05, two-way ANOVA followed by Newman-Keuls *post hoc* test. N = 6-8/group.

In the open field, all treatment groups showed marked increases in locomotor activity at the beginning of monitoring, with gradual habituation to the novel environment (Fig 3A and 3G). To investigate altered anxiety in the task, we measured the duration of time spent in the open area, and found no significant differences between treatment groups (Fig 3B and 3H). To determine anxiety-related phenotypes more closely, we subjected the animals to the light-dark box (Fig 3C–3F and 3I–3L). While having a common preference for staying on the dark side of the box, rodents have an innate drive to explore the open illuminated area. Unexpectedly, P7-9 sham animals did not exhibit such a preference, spending equal time in both the light and dark compartments. Most importantly, animals exposed to maternal separation or isoflurane treatments during P7-9 showed indistinguishable behavior compared to that of the sham group in the light-dark box test. In P15-17 mice, animals treated with isoflurane showed more rearings, as well as time spent and distance moved in the light zone of the box (Fig 3J–3L). Such a phenotype may indicate reduced anxiety, impulsiveness, and/or increased risk-taking. Both the isoflurane group and the maternal separation group also showed overall shorter latency to enter the light zone, but no significant difference was observed (Fig 3I).

**Fig 4:**
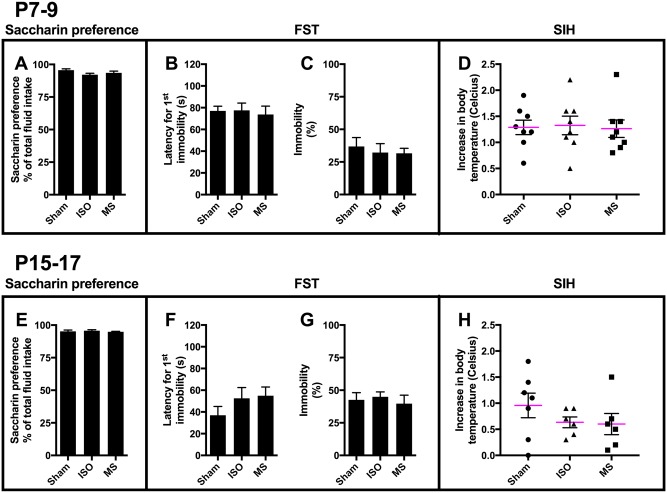
Early-life exposure to three consecutive and brief isoflurane anesthesias or maternal separations do not produce long-lasting depression-related behavior. Saccharin preference (**A, E**). Latency to immobility (**B, F**) and relative immobility during 2–6 min (**C, G**) in forced swimming test. Stress-induced hyperthermia (**D, H**). Abbreviations: ISO, isoflurane; MS, maternal separation; FST, forced swimming test; SIH, stress-induced hyperthermia. N = 6-8/group.

Saccharin preference test [30] and forced swim test [31] were used to assess depression-related endophenotypes. All mice displayed a strong preference to consume sweetened solution over regular tap water, and there were no significant differences across all treatment groups (Fig 4A and 4E). Animals subjected to isoflurane and maternal separation showed similar behavioral performance on the forced swim test as the sham animals (Fig 4B, 4C and 4F–4G). Hyperthermia in response to an acute stressor was also indistinguishable between the treatment groups (Fig 4D and 4H). Thus, repeated exposure to a brief isoflurane anesthesia or maternal separation during early postnatal development did not bring anhedonia (i.e. reduced consumption of sweetened solution), any obvious changes in “behavioral despair” or physiological response to acute stress in adult animals.

**Fig 5:**
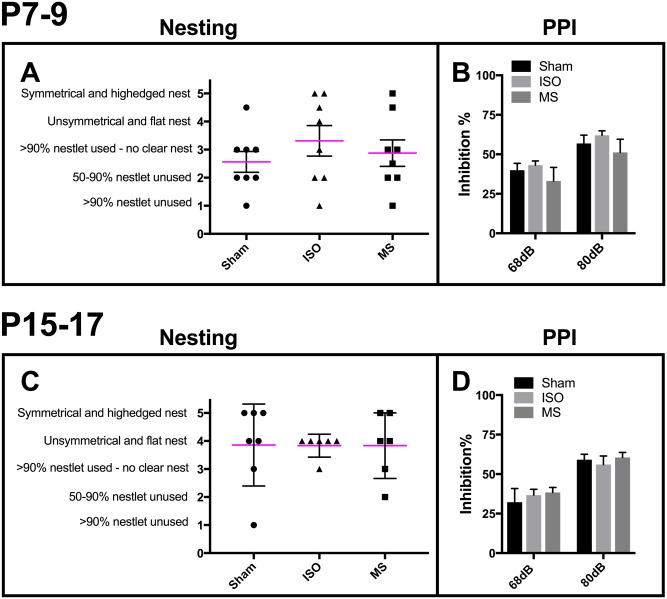
Early-life exposure to three consecutive and brief isoflurane anesthesias or maternal separations do not produce long-lasting schizophrenia-related behavior. Nesting behavior (**A, C**). Prepulse Inhibition (prepulse 68dB or 80dB) (**B, D**). Abbreviations: ISO, isoflurane; MS, maternal separation; PPI, prepulse inhibition. N = 6-8/group.

Anesthetics have been shown to produce a schizophrenia-like phenotype when administered during early postnatal development. Although such effects are clearly demonstrated with NMDA-receptor antagonists [32], GABA_A_ acting agents have also been shown to bring similar alterations, particularly in males [33–35]. Locomotor hyperactivity, abnormal social behavior and impaired nesting, and pre-pulse inhibition (PPI) are considered as “markers” of a schizophrenia-type phenotype in rodents [26,36,37]. Altogether, nesting behavior showed marked within-group variation (Fig 5A and 5C) making it difficult to judge potential differences across groups. Following PPI, which is one of the most translationally valid behavioral tests assessing psychiatric traits in animals, the pre-pulse significantly reduced tone-induced startle response in all animals (Fig 5B and 5D). These data, in combination with the lack of locomotor hyperactivity (Fig 3A and 3G) suggests that brief exposure to repeated isoflurane anesthesia at selected postnatal periods do not promote the expression of schizophrenia-like behavioral abnormalities in adult mice.

**Fig 6:**
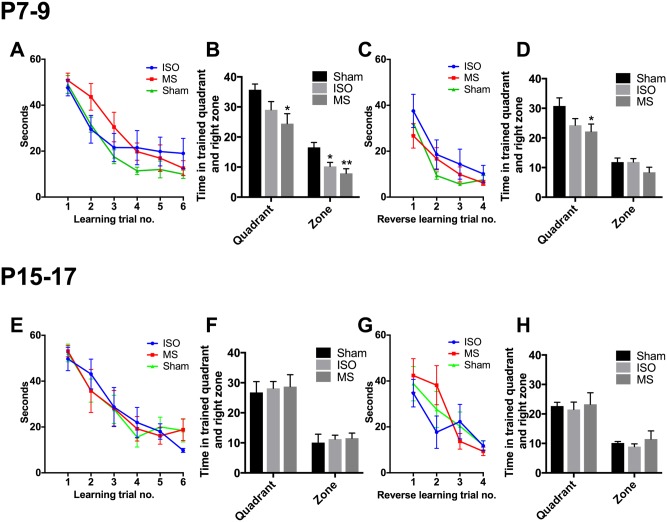
Early-life exposure to three consecutive and brief isoflurane anesthesias or maternal separations at postnatal days 7–9 bring mild deficit in spatial navigation memory. Latency to escape (find the platform) (**A, E**) and time spent near the vicinity of pre-existing platform (quadrant, zone) (**B, F**) during the first learning trials and probe test, respectively. Latency to escape (find the platform) (**C, G**) and time spent near the vicinity of pre-existing platform (quadrant, zone) (**D, H**) during the reverse learning trials and probe test, respectively. Abbreviations: ISO, isoflurane; MS, maternal separation. *<0.05, **<0.01, two-way ANOVA followed by Newman-Keuls *post hoc* test. N = 6-8/group.

To date, the most well documented side-effect associated with early-life anesthesia exposure in rodents (and humans) is reduced cognitive ability, including deficits in spatial navigation and learning and memory processes [10–12]. Here, we utilized the water maze, a standard test to measure spatial navigation, to assess whether brief anesthesia exposure during P7-9 or P15-17 produce similar cognitive deficits. All mice successfully completed this learning task without showing abnormal thigmotaxis behavior (data not shown), and performance became significantly more efficient on a trial-by-trial basis. However, both the isoflurane treated animals and animals subjected to maternal separation showed a tendency for longer escape latencies when exposed to the treatments at P7-9 (Fig 6A). The time spent around the vicinity of the trained platform was significantly reduced in isoflurane treated animals during the probe trial (Fig 6B). Similar behavior patterns were also observed in the maternal separation group. Furthermore, both isoflurane and maternal separation groups seem to perform less efficiently during the reverse learning, with time spent in the vicinity of the platform area significantly reduced in the maternal separation group (Fig 6C and 6D). Therefore, the potential learning disability can be hardly be explained by isoflurane-induced neuroapoptosis or cognitive deficits. The mice that were subjected to repeated anesthesia or maternal separation at postnatal days 15–17 showed no notable differences during the water maze test compared to the sham group. (Fig 6E–6H).

## Discussion

General anesthesia during early postnatal development has been shown to produce neuroapoptosis, altered synaptogenesis, and long-lasting behavioral dysfunctions in animals [12–16,18,38]. Majority of these studies have been conducted during the first two postnatal weeks in rodents, and with most experiments the duration of anesthesia has been a few hours or more. Given the radical differences in the timing of developmental events between mice and men, it is difficult to correlate and directly translate these findings to human conditions. A recent study utilized a shorter duration of general anesthesia in rats during different stages of postnatal development [8]. Already this 30-minute anesthesia induced neuroapoptosis and synapse loss when administered at P7, but these neuropathological changes did not persist into adulthood and did not lead to long-lasting behavioral abnormalities [8]. Interestingly, essentially the same anesthesia exposure produced a transient increase in synaptic density when delivered at P15, without leading to long-lasting behavioral consequences [8].

Since repeated exposure to anesthesia during early development may be more detrimental than a single exposure, we decided to investigate the effects of such a paradigm by assessing behavior in the adult mouse. The experiments were conducted with male mice, since previous studies have shown that male rodents are more susceptible to early-life anesthesia induced neuropathological and behavioral deficits when compared to females [23,24]. Specifically, we exposed male mouse pups to 30-minute isoflurane anesthesia or maternal separation on three consecutive days at two distinct developmental time points closely matching with the study by Qiu et al [8]: P7-9 or P15-17. We focused our studies on isoflurane, since it has been shown in several animal studies to produce prominent neuropathological and behavioral alterations [38–41]. Notably, isoflurane is also among the most widely used general anesthetics in preclinical animal research.

Isoflurane exposure during first postnatal week in rodents has been consistently associated with increased neuroapoptosis in hippocampal and thalamic structures as evidenced by caspase-3 activation and/or FluoroJade staining [12,15,40,42]. The long-term behavioral consequences of this have been extensively studied, but the findings have been inconsistent. Studies have found that both a 6-hour exposure to a mixture of midazolam, nitrous oxide, and isoflurane, as well as a 4-hour exposure to isoflurane at age P7 cause long-term impairments in spatial learning and memory or novel object recognition and social memory in rats [12,15,40]. On the contrary, a study by Loepke et al. [42] found that a 6-hour isoflurane-anesthesia in mice at P7 does not cause behavioral deficits in adulthood. Sevoflurane-induced impairment of spatial learning and memory in adulthood has been shown to be dependent on both the concentration and exposure duration of the anesthesia [19]. We utilized an isoflurane treatment paradigm known to produce deep burst suppressing (surgical) anesthesia in adult rodents [22]. Unexpectedly, the treatments did not cause gross phenotypic alterations in adult behavior when delivered at P7-9. Animals following isoflurane treatment showed only subtle deficits in spatial learning and memory when tested on the water maze. This phenotype was also present in the maternal separation group.

Interestingly, several GABA_A_ acting anesthetics (propofol, isoflurane, sevoflurane) have been shown to induce synaptogenesis and increase dendritic spine density in rodent hippocampus and medial prefrontal and somatosensory cortex already after a single 30-minute and up to 6-hour exposure when administered at a later stage of brain growth spurt during P15-16 [6–8,43]. These observations imply a fundamental change taking place in neuronal responses to anesthetics during early brain development. This may be due to a gradually occurring developmental shift of GABA_A_ -mediated responses on neuronal excitability (from depolarization to inhibition), caused by alternating changes in the expression of Na^+^/K^+^/Cl^-^ cotransporter NKCC1 and chloride-extruding K^+^/Cl^-^ cotransporter KCC2 [33,44].

Similarly to anesthesia-induced neuroapoptosis at first postnatal week, increased dendritic spine density after a brief anesthesia exposure at P15 does not persist into adulthood [8]. Long-lasting changes in synaptic morphology have been associated only with longer exposure times to general anesthesia [7,16]. The most pronounced behavioral alterations in animals treated at P15-17 were observed in the isoflurane-treated group, as characterized by hyperactive behavior in homecage activity and increased risk-seeking behavior in the light-dark box test. However, these behavioral alterations were modest.

In conclusion, the present study strongly indicates that brief general anesthesia, shown to elicit alterations in neuroapoptosis and neuronal integrity [8,42], produces differential yet mild behavioral changes in adult male mice when delivered at either P7-9 or P15-17. Longer exposure times to anesthesia or different combinations of anesthetics are therefore likely needed to produce gross behavioral deficits still evident in adulthood. Indeed, several early observations have found that a single brief anesthesia administration during early development causes transient neurobiological effects and negligible behavioral effects in adult rodents [8,42,43]. To further evaluate the translational impact of our current study, subsequent investigations that utilize different anesthesia protocols, animal species, developmental time points during anesthesia and relevant factors present in a clinical environment, including concomitant surgical operations, are needed [17].
